# Legends of Allergy—Lawrence B. Schwartz

**DOI:** 10.1111/all.70290

**Published:** 2026-03-02

**Authors:** Gunnar P. Nilsson

**Affiliations:** ^1^ Division of Immunology and Respiratory Medicine, Department of Medicine Solna, Karolinska Institutet, and Clinical Immunology and Transfusion Medicine Karolinska University Hospital, Solna, and Center for Molecular Medicine, Karolinska University Hospital Solna Sweden

**Keywords:** allergy diagnosis, biomarkers, mast cells

Many researchers and clinicians working with allergy, mastocytosis and other mast cell‐associated diseases have much to be grateful to Lawrence (Larry) Schwartz for (Figure 1). His groundbreaking discoveries in the late 20th century laid the foundation for our understanding of human mast cells (Box 1). By enabling the identification of distinct human mast cell subsets by immunohistochemistry and by quantifying mast cell enzymes, for example, tryptase, his work bridged basic science and clinical medicine. These advances profoundly influenced the diagnosis and management of mast cell‐driven diseases, including anaphylaxis and systemic mastocytosis.

**FIGURE 1:**
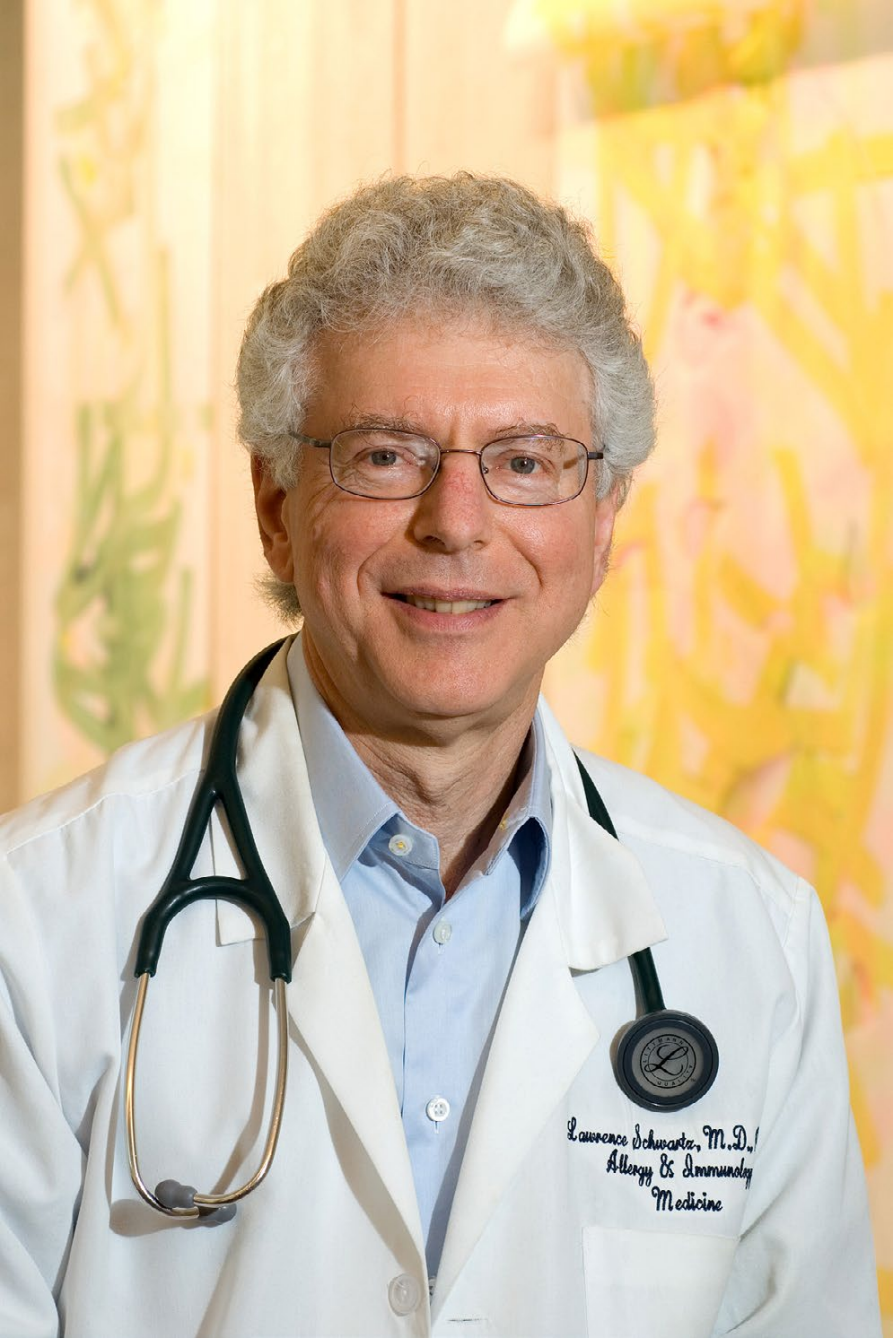
Larry Schwartz.

BOX 1 **Major contributions.**

1

**Table:** 

1979	Discovered that β‐hexosaminidase serves as a biomarker for mast cell degranulation in vitro
1981–2011	Purification, characterization, naming, cloning, expression, and processing of human α and β tryptases were accomplished.
1986	Two subsets of human mast cells with distinct protease compositions and tissue locations identified and characterized.
1986	Invention of commercial tryptase assay(s) using anti‐tryptase mAbs that facilitated the diagnosis of anaphylaxis, systemic mastocytosis and other mast cell disorders.
1992	Demonstration that rhuSCF stimulates the development of human mast cells from progenitor cells in human fetal liver and cord blood.
2019	Discovery of naturally occurring α/β‐tryptase heterotetramers that uniquely activate protease‐activated receptor 2 and prime the mechanosensory receptor called EMR2.

Larry completed a training "program" at Washington University, St. Louis, Missouri, where he also completed medical school and a residency in Internal Medicine. His interest in mast cells and their proteases began after his move in 1978 to Robert B. Brigham Hospital and Harvard Medical School, Boston, MA. There he joined the department of the legendary K. Frank Austen as a fellow in Rheumatology and Allergy/Immunology.

The following 5 years laid the groundwork for his lifelong career in mast cell biology. Among his early discoveries, sometimes overshadowed by later achievements, was the finding that mast cells release the enzyme beta‐hexosaminidase upon degranulation [1]. Measurement of beta‐hexosaminidase has since become a widely used method for assessing mast cell degranulation in vitro. Probably his most important accomplishment during this period was the purification of human mast cell protease tryptase, marking the beginning of his remarkable contributions to human mast cell research [2].

In 1983, Larry was appointed Associate Professor of Internal Medicine at Virginia Commonwealth University (VCU), Richmond, VA, and became full Professor in 1989. It was at VCU that the majority of his most influential work was accomplished. Soon after establishing his laboratory, he generated monoclonal antibodies against human tryptase and chymase, tools that remain widely used today.

Using these antibodies, he made two seminal discoveries. First, he identified two distinct human mast cell subtypes: those expressing only tryptase (designated MC_T_), found primarily in mucosal epithelium, and those expressing both tryptase and chymase (MC_TC_) located mainly in subepithelial and parenchymal tissues (Figure 2) [3]. This classification remains valid today, even as it is being challenged in the era of single mast cell RNA‐sequencing. Second, he demonstrated that tryptase levels in serum and plasma are elevated acutely during hypotensive anaphylactic reactions or at baseline in most patients suffering from systemic mastocytosis [4]. This work laid the foundation for the use of tryptase as a biomarker of mast cell activation and/or mast cell burden. Today, tryptase immunoassays are routinely used to help diagnose systemic mastocytosis and anaphylaxis and have also contributed to the discoveries and diagnoses of mast cell activation syndromes (acute elevations of serum tryptase) and of hereditary alpha‐tryptasemia (elevated baseline tryptase), as well as for monitoring of mast cell cytoreductive therapies.

**FIGURE 2:**
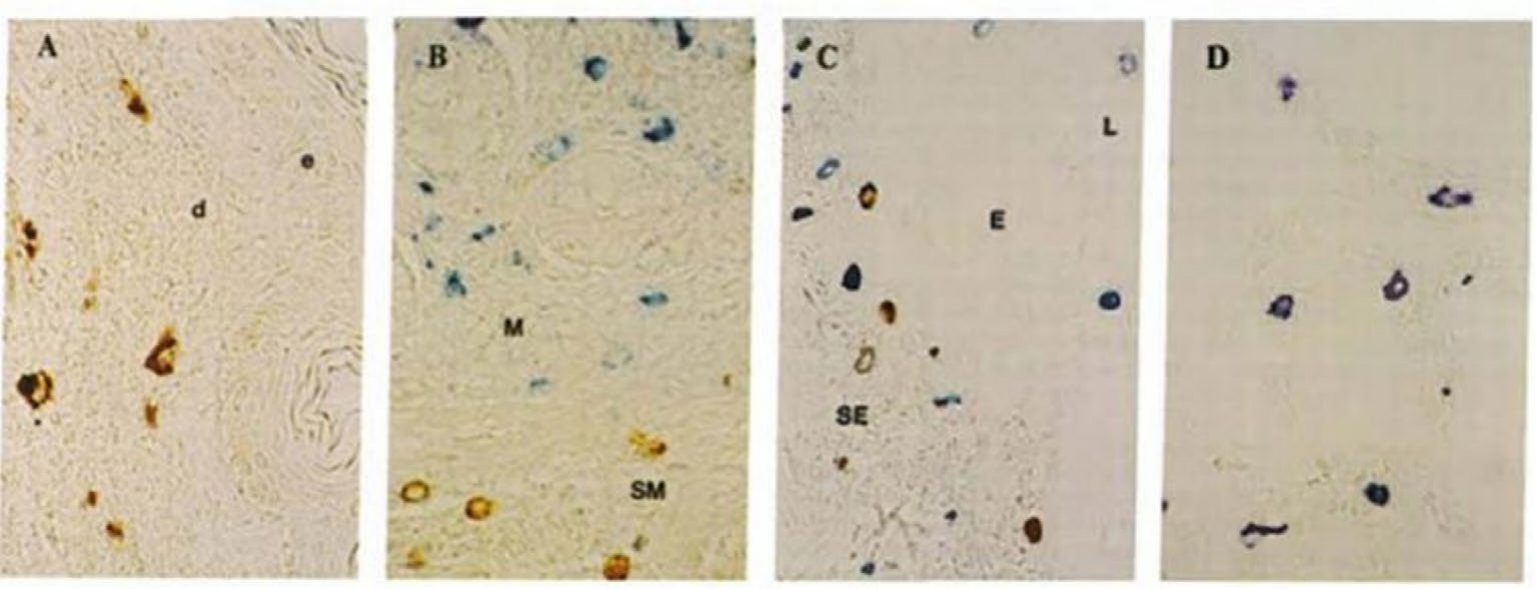
Seminal immunohistochemical description of two distinct human mast cell subtypes using monoclonal antibodies against tryptase and chymase [3]. Those that express only tryptase (MC_T_) (blue) and those that express both tryptase and chymase (MC_TC_) (brown). Sections of skin (A), small Bowel (B), bronchus (C), and alveoli (D).

With his continued interest in the biochemistry of proteases, Larry has continued to characterize their proteolytic activities. One of his most recent findings involves hereditary alpha‐tryptasemia, a genetic trait with a duplication of the alpha‐tryptase gene, where he and collaborators demonstrated that this alters enzyme specificity [5]. In addition, it is worth mentioning that his laboratory, at the same time as three other laboratories in the US and Austria, demonstrated that stem cell factor is the most important growth factor for the development of human mast cells [6].

Larry is truly a legend in the field of allergy and mast cell biology. Beyond his extraordinary scientific contributions, he has been a generous and inspiring mentor to researchers and clinicians. Most importantly, his passion and dedication have made a real difference for patients living with systemic mastocytosis, anaphylaxis, and other mast cell‐driven diseases. His discoveries continue to shape contemporary practice, advancing precision diagnostics and enabling more personalized management of mast cell‐mediated disorders.

## Funding

The author has nothing to report.

## Conflicts of Interest

The author declares no conflicts of interest.

## Data Availability

The author has nothing to report.

## References

[1] L. B. Schwartz , K. F. Austen , and S. I. Wasserman , “Immunologic Release of Beta‐Hexosaminidase and Beta‐Glucuronidase From Purified Rat Serosal Mast Cells,” Journal of Immunology 123 (1979): 1445–1450. 479592

[2] L. B. Schwartz , R. A. Lewis , and K. F. Austen , “Tryptase From Human Pulmonary Mast Cells: Purification and Characterization,” Journal of Biological Chemistry 256 (1981): 11939–11943. 7028744

[3] A. A. Irani , N. M. Schechter , S. S. Craig , G. DeBlois , and L. B. Schwartz , “Two Types of Human Mast Cells That Have Distinct Neutral Protease Compositions,” Proceedings of the National Academy of Sciences of the United States of America 83 (1986): 4464–4468. 3520574 10.1073/pnas.83.12.4464 PMC323754

[4] L. B. Schwartz , D. D. Metcalfe , J. S. Miller , H. Earl , and T. Sullivan , “Tryptase Levels as an Indicator of Mast‐Cell Activation in Systemic Anaphylaxis and Mastocytosis,” New England Journal of Medicine 316 (1987): 1622–1626. 3295549 10.1056/NEJM198706253162603

[5] Q. T. Le , J. J. Lyons , A. N. Naranjo , et al., “Impact of Naturally Forming Human Alpha/Beta‐Tryptase Heterotetramers in the Pathogenesis of Hereditary Alpha‐Tryptasemia,” Journal of Experimental Medicine 216 (2019): 2348–2361. 31337736 10.1084/jem.20190701 PMC6780998

[6] A. A. Irani , G. Nilsson , U. Miettinen , et al., “Recombinant Human Stem Cell Factor Stimulates Differentiation of Mast Cells From Dispersed Human Fetal Liver Cells,” Blood 80 (1992): 3009–3021. 1281684

